# Silver Nanoparticles: Synthetic Routes, In Vitro Toxicity and Theranostic Applications for Cancer Disease

**DOI:** 10.3390/nano8050319

**Published:** 2018-05-10

**Authors:** Valeria De Matteis, Mariafrancesca Cascione, Chiara Cristina Toma, Stefano Leporatti

**Affiliations:** 1 Dipartimento di Matematica e Fisica “E. De Giorgi”, Università del Salento, Via Monteroni, 73100 Lecce, Italy; chiara.toma@unisalento.it; 2 Dipartimento di Scienze Biomediche e Oncologia Umana, Università degli Studi di Bari “Aldo Moro”, p.zza G. Cesare, 70124 c/o Policlinico Bari, Italy; mariafrancesca.cascione@unisalento.it; 3 CNR Nanotec-Istituto di Nanotecnologia, c/o Campus Ecotekne, Via Monteroni, 73100 Lecce, Italy

**Keywords:** silver nanoparticles, synthetic routes, toxicity, theranostics

## Abstract

The large use of nanomaterials in many fields of application and commercial products highlights their potential toxicity on living organisms and the environment, despite their physico-chemical properties. Among these, silver nanoparticles (Ag NPs) are involved in biomedical applications such as antibacterial agents, drug delivery vectors and theranostics agents. In this review, we explain the common synthesis routes of Ag NPs using physical, chemical, and biological methods, following their toxicity mechanism in cells. In particular, we analyzed the physiological cellular pathway perturbations in terms of oxidative stress induction, mitochondrial membrane potential alteration, cell death, apoptosis, DNA damage and cytokines secretion after Ag NPs exposure. In addition, their potential anti-cancer activity and theranostic applications are discussed.

## 1. Introduction

The unique physico-chemical properties of silver nanoparticles (Ag NPs) have attracted increasing interest from the scientific community [1] due to their high thermal conductivity, plasmonic properties, chemical stability and antibacterial ability [2]. The use of Ag is not new; it dates to the times of Hippocrates, who used it as antibacterial to manage ulcers [3]. Nowadays, Ag NPs are used in many commercial products including soaps, plastics, food, textiles, catheters, and bandages. It is calculated that around 383 products are based on nano Ag globally, corresponding to 24% of all nano-products in use. For this reason, researchers are attempting to understand the adverse effects on living organisms: numerous in vitro and in vivo studies have demonstrated their toxicity occurring by means of cellular pathway perturbations [4]; nevertheless, their mechanism of action is still unclear. A lot of factors (size, shape, morphology, surface chemistry, charge, coating/capping agents, agglomeration, purity) influence the biological activity of Ag NPs and as consequence the adverse effects are different in different cell types [5].

Another important feature of Ag NPs is their involvement in cancer treatment. Cancer is a disease characterized by uncontrolled cellular growth and spread, during which cells become unresponsive to the usual check-points, leading to tumor growth and metastasis [6]. However, chemotherapy does not specifically target drugs to cancerous sites, therefore exposing healthy cells to undesirable effects. Moreover, a large dose is required owing to its rapid elimination and nonspecific distribution [7]. For these reasons, the goal of nanomedicine is to identify cost-effective molecules that have high specificity and sensitivity in cells **[8]**. With this in mind, Ag NPs are a promising tool as anticancer agents in diagnostics and probing [9], with strong effects against different cancer cell lines offering many advantages [10]. Their better penetration, and the possibility to track Ag NPs in the body make them a more efficient tool in cancer treatment with less risk compared to standard therapeutic procedures [11]. The unique Ag NP properties, such as easy surface functionalization, optical properties, reproducible synthetic routes and high surface: volume ratio, makes them suitable for cancer treatment [12]. The optical properties can be tuned to have an absorption at specific wavelengths that is useful for imaging and photothermal applications in tissue [13]. Ag NPs can also be functionalized with different molecules such as DNA/RNA to selectively target different cells [14] and antibodies or polymers [15]. These last agents are important to extend the half-life time for in vivo circulation that is critical in drug and gene delivery applications [16]. In addition, Ag NPs are used as an ablation tool for cancer cells due their ability to convert radiofrequencies into heat [17]. 

Starting from these assumptions, in this review we focused on the synthetic routes used for the synthesis of Ag NPs (physical, chemical and green methods), the mechanism of their toxicity in cells and their potential applications in theranostics for cancer.

## 2. Principal Routes of Ag NPs Synthesis

Engineering nanoparticles (NPs) can be synthetized by different approaches: “top-down”, and “bottom-up”. The first means the nanoparticle synthesis comes from a bulk system, while bottom-up means they are obtained from nanoscaled materials starting from atomic level. The top-down is exploited in synthesis based on physical methods, whereas the bottom-up approach is useful for chemical or green procedures, and it is more suitable for obtaining monodispersed nanostructures with fewer defects [18].

### 2.1. Physical Synthesis

The use of physical methods allows the synthesis of nanoparticles in the absence of solvent, permitting the uniformity of Ag NPs size distribution compared to chemical routes [19]. 

The main routes of synthesis are listed below:

- Evaporation-condensation

One of the common physical routes is the evaporation-condensation method, using a tube furnace at atmospheric pressure, where the source material is centered in a furnace and vaporized into a carrier gas. Many types of NPs are synthetized using this technique, including Ag NPs [20]. However, this procedure presents many limitations, among them high consumption of energy, large time to achieve the operating temperature. Together with bulky shape instrumentation, this makes this technique of little use.

- Laser ablation

**Figure 1:**
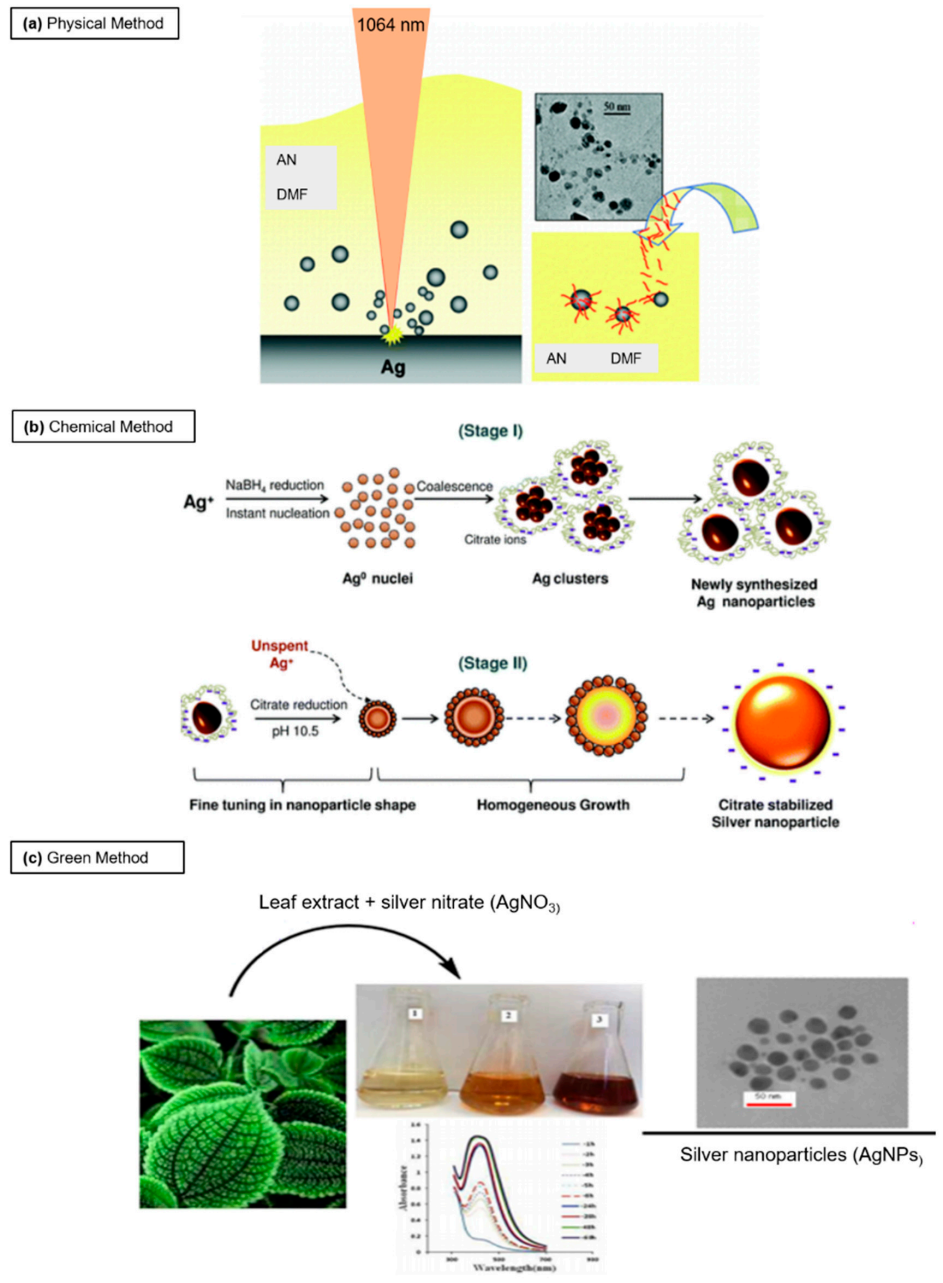
Schematic representation of common routes to synthetize Ag NPs with different approaches: (**a**) physical approach (laser ablation) adapted from [24] with permission from American Chemical Society, Copyright 2007: a laser pulses of 1064 nm focused with a 10 cm focus lens on Ag plate in pure acetonitrile (AN) and *N*,*N*-dimethylformamide (DMF) was used in order to obtain Ag NPs with a size of 5 nm, (**b**) chemical approach (chemical reduction), adapted from [45] with permission from The Royal Society of Chemistry, 2014: Ag NPs were obtained from a solution of AgNO_3_ using NaBH_4_ as a primary reductant and trisodium citrate, both as secondary reductant as well as stabilizing agent, and (**c**) green approach (plant extract) adapted from [92] with permission from The Royal Society of Chemistry, 2016: Ag NPs (20–30 nm) were synthetized by adding *Ferula latisecta* leaves extract to 1 mM aqueous AgNO_3_ solution. The solution turned from yellow to dark brown.

Ag NPs can be produced by laser ablation method, which permits the obtaining of uncontaminated colloids, even if the control of size is difficult. This method uses a laser beam as the energy source to induce ablation on a solid target material, which vaporizes into atoms and clusters, and successively the NPs are assembled in ambient media (gas or liquid) [21]. Pyatenko et al. [22] synthetized Ag NPs by a laser ablation, irradiating a bulk Ag with a 532 nm laser beam in pure water. They obtained 2–5 nm Ag NPs after use of high laser power and small spot sizes. Tsuji et al. [23] used focused and unfocused laser beam irradiation with two intensities (12 and 900 mJ/cm^2^ respectively). They demonstrated a reduction in terms of NP diameter (from 29 nm to 12 nm) with the decrease of laser wavelength. Amendola et al. [24] obtained stable solutions of Ag NPs in pure acetonitrile and *N*,*N*-dimethylformamide by laser ablation of the bulk metal with a size of (1.9 ± 1.5) nm and (2.2 ± 2.5) nm respectively (Figure 1a). 

- Thermal decomposition

Thermal decomposition is an endothermic chemical reaction induced by heat. The substances have a specific thermal decomposition that cause chemical decomposition. Therefore, the process permits the break-up of large molecules into smaller ones due to heat in an oxygen-restricted environment [25]. Ag NPs have also been synthesized using thermal decomposition method by Lee and Kang [26], using a solution of AgNO_3_ and sodium oleate. The synthesis of spherical Ag NPs with a size ranging from 9.5 nm to 0.7 nm was obtained by changing the temperature from room temperature to 290 °C for 1 h. Navaladian et al. achieved [27] spherical crystalline Ag NPs (10 nm) by thermal decomposition of Ag oxalate in water and in ethylene glycol, using polyvinyl-alcohol (PVA) as a capping agent, which reduces Ag oxalate. The Ag NPs became smaller when the PVA concentration increased. 

- Ultrasonic spray pyrolysis

Another physical approach is ultrasonic spray pyrolysis, which allows the obtaining of NPs with a controlled size through the production of aerosol from a dilute aqueous metal salt solution. Pluym et al. [28] produced pure and monodispersed micron-sized Ag NPs by spray pyrolysis with an ultrasonic generator at 600 °C (using N_2_ carrier gas), and above 900 °C (using air carrier gas). Pingali et al. [29] proposed a one-step synthesis of Ag NPs with a diameter ranging from 20 nm to 300 nm, by pyrolysis of ultrasonically atomized spray of AgNO_3_ solution, maintaining a temperature of 650 °C with a tube furnace. Different sizes were obtained tuning the concentration of AgNO_3_ solution and ultrasound power.

- Arc discharge method

Arc discharge method is mostly used to synthetize carbon nanotubes (CNTs): a current arc voltage is applied between two graphite electrodes that vaporize carbon in the presence of a catalyst immersed in an inert gas (helium or argon) [30]. Recently, this method is used for synthesis of metallic NPs such as Ag NPs.

Arc discharge method requires vacuum apparatus with an efficient cooling system. Tien et al. [31] used it to produce Ag NPs into ultrapure water. They adopted two Ag electrodes that, after melting at high temperature, were converted into Ag NPs with a size ranging from 5 nm to 45 nm. Another study [32] described the production of spherical Ag NPs (3–5) nm by direct metal sputtering into propane-1,2,3-triol (glycerol). 

Although physical methods were consistent for obtaining good Ag NPs without the use of hazardous chemicals, their use is often unfeasible due to the limitations of large energy consumption and long times required to reach thermal stability [33].

### 2.2. Chemical Approach

- Chemical reduction

The most used chemical approach is chemical reduction, which permits synthesis of Ag NPs in solution (water or organic solvents) in presence of (i) metal precursors, (ii) reducing agents sodium citrate, ascorbate, sodium borohydride (NaBH_4_), elemental hydrogen, polyol process, Tollens reagent, *N*,*N*-dimethylformamide (DMF), poly(ethyleneglycol)—block co polymer (PLGA-PEG-PLGA), and (iii) stabilizing/capping agents poly(vinyl alcohol) (PVA), poly(vinyl pyrrolidone) (PVP), poly(ethyleneglycol) PEG, poly(methacrylic acid) (PMAA), poly(methyl methacrylate) (PMMA) [31]. 

The reduction of Ag salts induces the formation of colloidal solutions: this process occurs in two steps (nucleation and growth) that strongly influence the shape and the size of Ag NPs. These two critical steps are controlled by pH, temperature, stabilizing agents and reduction agents [34,35]. Dadosh [36] developed a one-step synthesis of Ag NPs using tannic acid as reducing agent together with sodium citrate. The modification of tannic acid concentrations induced the formation of different sized (from 18 nm to 30 nm) Ag NPs. Based on this procedure, De Matteis et al. [37] optimized the synthetic route, in terms of AgNO_3_ concentration and temperature. Zhang et al. [38] used a hyper-branched poly(methylene bisacrylamide aminoethyl piperazine) with terminal dimethylamine groups (HPAMAM-N(CH_3_)_2_) to produce Ag NPs. Wang et al. [39] proposed the generation of Ag NPs using a solution of PVP and AgNO_3_ with glucose as reducing agent. The solution was completed with NaOH to increase the speed of reaction. The stability of solution took placed when the mole ratio of NaOH and AgNO_3_ varied between 1.4 and 1.6, without Ag^+^. Suriati et al. [40] obtained Ag NPs (35–80 nm) using trisodium citrate and ascorbic acid as surfactants. The increase of ascorbic concentration induced the formation of polygonal NPs with a large size while the size was reduced increasing the trisodium citrate amount, and the shape was quasi-spherical. The use of gallic acid as reducing and stabilizing agent was adopted to obtain 7, 29, and 89 nm of Ag NPs from AgNO_3_ [41]. The authors used two pH values (11 and 10) to obtain 7 and 29 nm respectively. Also, another method allowed the synthesis of Ag NPs (24 nm) by exploiting hydrazine hydrate and sodium citrate as reducing agents [42] and sodium dodecyl sulphate as stabilizing agent in presence of AgNO_3_ 8% (*w*/*w*). In other routes, tri-sodium citrate and NaBH_4_ acted as stabilizer and the reducing agent respectively, in presence of AgNO_3_ [43,44]. Agnihotri et al. [45] synthetized monodispersed Ag NPs (5, 7, 10, 15, 20, 30, 50, 63, 85, and 100 nm) with a co-reduction employing sodium borohydride (NaBH_4_) as a primary reductant and trisodium citrate (TSC), as secondary reductant and a stabilizing agent. NaBH_4_ allowed to form instant nuclei generation, while TSC was important to control the particles size (Figure 1b).

- Microemulsion

Microemulsion method is a reproducible technique that allows the obtaining of uniform size NPs and it is based on the use of three precursors: (i) polar phase that commonly is water, (ii) non-polar phase as hydrocarbon liquid or oil, and (iii) surfactant [46]. The two phases are separated by the presence of surfactant, which forms an interfacial layer, reducing the interfacial tension between the microemulsion, and inhibiting the coalescence of the droplets. This technique (water-in-oil (w/o) or oil-in-water (o/w) depending on the surfactant) allows the obtaining of monodispersed and stable Ag NPs using two microemulsions: one with water core of AgNO_3_ (as Ag^+^ source) and the other water core with hydrazine hydrate (as reducing agent) [47,48]. The reaction starts with the addition of non-toxic dodecane (oil phase) and sodium bis (2-ethylhexyl) sulfosuccinate (AOT, surfactant). Pileni et al. [49] used w/o droplets to tune the size of Ag NPs that were extracted from reverse micelles and mixed in nonpolar solvent. They found that the diameter of NPs changed from 2 nm to 7 nm, after increasing the size of water drops. Chen et al. [50] reduced AgNO_3_ with AOT, obtaining cubic shaped Ag NPs, with diameters less of 10 nm. 

- Sonochemical method

Many NPs of different materials were synthetized by sonochemical method, which have the advantages of eliciting rapidity of reaction time and formation of small NPs. The method includes three steps: formation, growth and implosive collapse of microcavities [51]. Ultrasound waves perturb chemical reactions derived from high temperature (5000 K) and pressure (1000 atm), inducing the collapse of microscopic bubbles (cavities), which expand during the decompression step and implode in the compression phase [52,53]. Gutierrez et al. [54] used this method to produce Ag NPs from a solution of Ag^+^ by sonication at 1 MHz under argon-hydrogen atmosphere. Wani and co-workers [55] synthetized Ag NPs using two reducing agents (sodium borohydride and sodium citrate) in two separate reactions irradiated with the same 20 KHz ultrasound waves. Spherical Ag NPs (10 nm) were obtained using NaBH_4_, while smaller NPs (3 nm) were formed in the presence of sodium citrate. 

- Sol-gel

The sol-gel process is a colloidal chemistry technology, which is widely applied to synthetized metal and metal oxide NPs at low temperature. In the first phase, the monomers of materials are converted into a colloidal solution (sol), which represents the precursor (metal alkoxides or chlorides) for gel formation, which in turn formed particles or polymers. To obtain colloids, the precursors are hydrolysed and polycondensed [56].

Zhai et al. [57] used a sol-gel route to synthetize Ag NPs embedding hybrid materials with organicosilica precursors (tetraethoxysilane, mercaptopropyltrimethoxysilane and polymethylhydrosiloxane) in alcoholic conditions. The three precursors act as framework constructor, a complexing agent toward metal ions and an in situ reducing agent for Ag^+^. Lkhagvajav et al. [58] prepared Ag NPs using AgNO_3_ and glucose as a reducing agent, which were first mixed in distilled water. The formation of NPs was showed when the solution become transparent.

- Electrochemical synthetic method

Electrochemical synthetic method is used to synthetize Ag NPs with a controllable size. In fact, the size can vary by electrolysis parameters modifications, while the monodispersion grade depends on the electrolytic compositions [31]. The detailed protocol of this method was described by Reetz and Helbig [59]. The authors reported Ag NPs synthesis in which a metal sheet was anodically dissolved, thus the formed metal was reduced at the cathode, giving rise to metallic particles stabilized by tetra alkylammonium salts. Ma et al. [60] obtained spherical Ag NPs (10–20 nm) in aqueous solution, choosing PVP to stabilize the Ag clusters and to reduce agglomerations. The application of rotating platinum cathode permitted the obtaining of a high monodispersity route, while the sodium dodecyl benzene sulfonate enhanced particle size distribution. This method was also adopted for the synthesis of the Ag NPs in acetonitrile containing tetrabutylammonium salts [61].

The possibility of obtaining monodisperse NPs with a tunable size is an advantage in the use of chemical methods, together with low cost and rapidity of steps [33]. However, the disadvantages of these methods are the hazardous and toxic elements employed in the synthetic routes and difficulty in purification. Although chemical and physical methods are considered suitable methods to synthetize well-defined nanoparticles, the scientific community is moving towards green synthesis of NPs.

### 2.3. Green Synthesis

The use of natural products as a source of reagents for NP synthesis is defined as green synthesis [62]. The natural source useful for synthetized Ag NPs can be divided in three categories:

(a) microorganisms (fungi, yeasts, bacteria, and actinomycetes),
(b) plants and plant extract,
(c) membranes, viruses’ DNA, and diatoms.

Microorganisms are able to produce metallic NPs by enzymes that contribute to physiological cellular activities. Depending on the NPs localization, the synthesis can take place intracellularly or extracellularly [62]. The intracellular synthesis of Ag NPs are due to the transfer of metal ions into the microorganism, while the extracellular route includes the localization of metal ions onto the cell surface. The enzymes, functional groups, proteins and enzymes are designated to reduce ions [63].

- *Bacteria*

Bacillus licheniformis was used to synthetized 50 nm Ag NPs by Kalimuthu et al. [64] in the presence of an aqueous solution of AgNO_3_, with enzyme nitrate as a stabilizer. Culture supernatants of *Staphylococcus aureus* as well as supernatants derived from *Enterobacteriaceae* were exploited to synthetize Ag NPs [65]. Many lactic acid bacteria (*Lactobacillus* spp., *Pediococcus pentosaceus* and *Enterococcus faecium*) have the ability to produce glucose, galactose, mannose and fructose, thus they are involved in redox reactions to obtain Ag NPs [66]. Ag NPs with different physico-chemical properties were also synthetized from *Escherichia coli* [67], *Klebsiella pneumonia* [68], *Pseudomonas stutzeri* [69], *Brevibacterium casei* [70], *Bacillus megaterium* [71], *Proteus mirsabilis* [72], *Plectonema boryanum* [73], and *Enterobacter cloacae* [74].

- *Fungi*

Fungi are considered useful tools for Ag NPs synthesis, due to their ability to quickly collect and uptake metals as well as the easy set-up equipment in the laboratory compared to bacteria. The large number of enzymes produced by fungi allow the reduction of AgNO_3_ solution on their surface [75]. Bhangale et al. [76] used biomass derived from *Aspergillus flavus* for the synthesis of spherical Ag NPs with a size of 7.13 nm. Vigneshwaran and co-workers [77] reported a similar synthesis of NPs with a size of (8.92 ± 1.61) nm. *Penicillium fellutanum* produces the enzyme nitrate reductase in culture filtrate, which was involved in reduction of Ag^+^ [78]. At the same time, the fungus *Aspergillus niger* was used to produce Ag NPs extracellularly [79]. In this case, the combination of reductase enzyme and quinine induced a transfer of electrons extracellularly [80]. In some cases, Ag NPs are not obtained in the solution, but on the surface of fungus where Ag+ are confined. This phenomenon is probably induced by the presence of wall proteins with carboxylate groups that have negative charge. In addition, the proteins contributed to the Ag nuclei formation that increase flowing the Ag^+^ reduction [81]. Ag NPs are also derived from fungi as *Aspergillus terreus* [82], *Bryophilous rhizoctoni* [83], *Pleurotus ostreatus* [84], *Aspergillus flavus* [77].

- *Plants*

**Figure 2:**
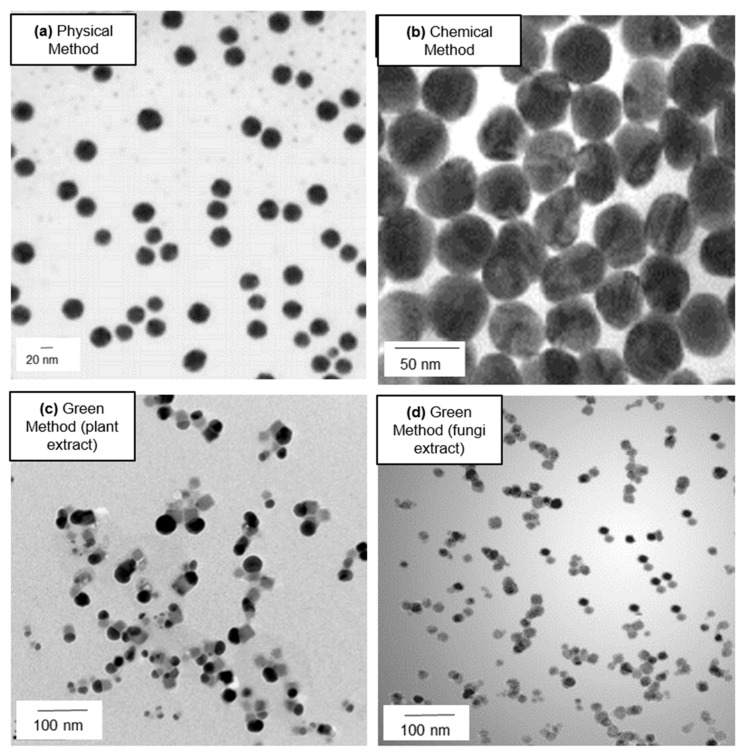
Representative TEM images of Ag NPs synthetized by different synthetic routes: (**a**) Ag NPs (9.4 ± 5.9) nm obtained by 532 nm laser ablation of a Ag rod using 60 mJ/pulse at 1.0 × 10^−3^ M of CTAB. Adapted/reprinted from [96], Copyright (2002), with permission from Elsevier; (**b**) Ag NPs (130 nm) produced by chemical method with ascorbic acid reduction at pH 10. Adapted/reprinted from [97], Copyright (2010), with permission from Elsevier; (**c**) Ag NPs (from 3 nm to 44 nm) synthesized from fresh *Codium capitatum* extract. Adapted from [98], Creative Commons Attribution License (CC BY) (**d**) Ag NPs (5–25) nm obtained from *Penicillium fellutanum Biourge*. Adapted from [78] with permission of Elsevier, 2009.

The non-toxic and eco-friendly method of synthetizing NPs is biosynthesis, which includes the reduction of Ag^+^ by biomolecules (saponins, proteins, tannins, amino polysaccharides, enzymes, alkaloids, vitamins, etc.) from plant extracts [85]. In general, adding plant extracts to AgNO_3_ solution induces the reduction of Ag^+^, and this phenomenon can be visualized with Ultra-violet Visible (UV-VIS) spectroscopy in time points. Many portions of flora are employed to obtain Ag NPs. Extracts of *Alternanthera dentate* have permitted the rapid obtaining of spherical Ag NPs with a size ranging from 50 nm to 100 nm, with antibacterial activity [86]. Dried roasted *Coffea arabica* seed extract in the presence of AgNO_3_ induced a reduction of Ag^+^ showing a color changing from yellow to dark brown. By this approach, the nano Ag exhibited spherical (20 nm) and ellipsoidal (30 nm) shape [87]. Tribulus terrestris was used to obtain spherical Ag NPs (16–28 nm) due to the mix of extracts and AgNO_3_. Also, in this case, the NPs have strong antibacterial properties against *Escherichia coli*, *Staphylococcus aureus*, *Pseudomonas aeruginosa* and *Bacillus subtilis* [88]. Krishnaraj et al. synthetized spherical Ag NPs (15–50 nm) from the leaf extract of *Acalypha indica* [89]. Some researchers exploited orange peel (*Citrussinensis*) to obtain nanomaterials derived from food waste. They obtained spherical NPs with an average size of 6 nm [90]. Khalil et al. [91] used olive leaf to reduce AgNO_3_ in order to obtain Ag NPs. The authors observed a significant synthetic route with the increase of pH and temperature that contributed to spherical Ag NPs with a size of (20–25) nm. A concentration of (0.03–0.07) mg/mL of NPs induced several toxic effects against *Staphylococcus aureus*, *Pseudomonas aeruginosa*, and *Escherichia coli*. In a recent work, Rashid et al. [92] employed *Ferula latisecta* leaf extract and 1 mM-aqueous AgNO_3_ to obtain Ag NPs with a diameter of about 20 nm (Figure 1c). Green synthesis is a promising route for synthesis of biocompatible and stable nanoparticles that minimizes the waste and energy costs and reduces the toxicity in comparison with physical and chemical methods [93]. Indeed, this method is environmentally friendly, easily scaled up for large-scale syntheses of nanoparticles, and is low cost. In addition, in the synthetic routes do not involve high temperatures, pressures, energy or toxic chemicals [94]. However, some disadvantages should be considered: for example, in the case of plants, they produce low yield of secreted proteins, which decreases the synthetic rate [95], while the manipulation of bacteria presupposes the use of specific set-up that, in many cases, is expensive. Representative Transmission electron microscopy (TEM) images of Ag NPs obtained with different methods were showed in Figure 2a–d.

## 3. In Vitro Cytotoxicity Mechanism Induced by Ag NPs

**Figure 3:**
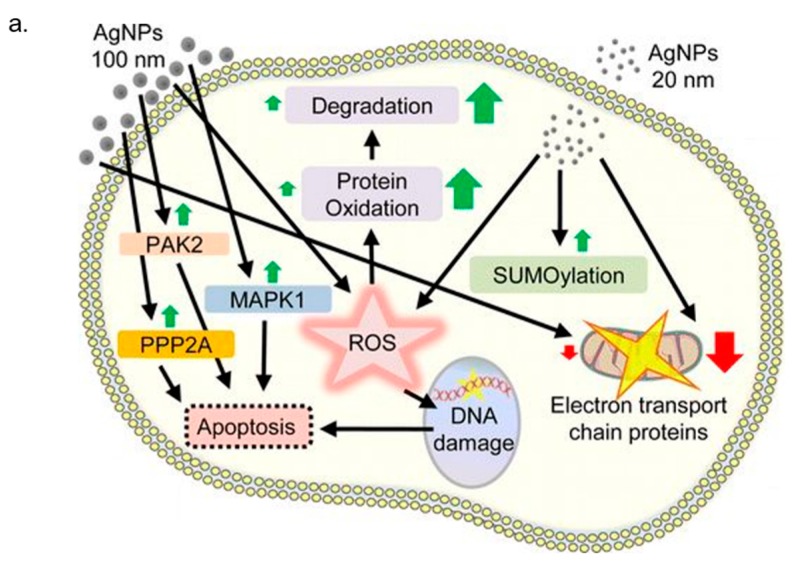

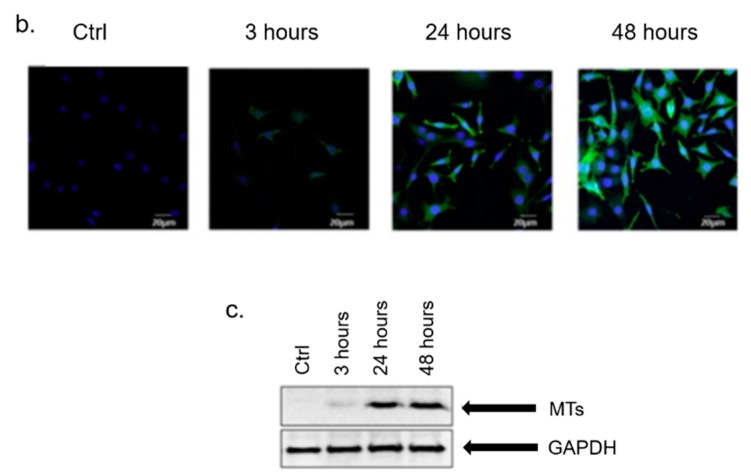
(**a**) Schematic representation mechanism of toxicity induced by AgNPs in cell. Reprinted/adapted with permission from [102], Copyright (2014) American Chemical Society (**b**) Representative images of Hela cell lines exposed to AgNPs at different time points and immunostained to detect metallothionein expression. Nuclei are stained by DAPI staining and metallothioneins with a primary anti-metallothioneins antibody coupled with a FITC-labeled secondary antibody. The expression of metallothioneins in cells is dependent on time. (**c**) Western blot analysis of metallothionein expression in HeLa cells treated with AgNPs. Adapted from [37] with permission from Elsevier, 2015.

The level of cytotoxicity relating to the rate of internalized NPs was influenced by their size; this evidence was confirmed in the Ag NPs case [99,100,101]. In particular, Liu and co-workers demonstrated the size-dependent cytotoxicity of Ag NPs (approximately 5, 20 and 50 nm) in terms of cell morphology, cell viability, cellular membrane integrity, oxidative stress and cell cycle progression, in four human cell lines (A549, SGC-7901, HepG2 and MCF-7). The obtained results, with quantified EC50 values, ensured a greater cytotoxic response to the nano Ag size [99]. Furthermore, as obtained by Hsin et al. [100], the decreased viability in NIH_3_T_3_ mouse fibroblast, A10 rat vascular smooth muscle and HCT116 human colon cancer cells was induced only from Ag particles having a size smaller than a 100 nm. Several studies reported in the literature suggest how the Ag NP toxicity occurs in sequential steps. When the Ag NPs were endocitated, they undergo a degradation process that induce a release of Ag^+^ causing Reactive Oxygen Species (ROS) generation and glutathione (SGH) level reduction. The augmentation of cellular superoxide radicals triggers the alterations in the transmembrane potential of mitochondria and influences the signal transduction pathways, which play an important role in apoptosis program activation and cell death [102] (Figure 3a). Carlson et al. evaluated alterations, in terms of mitochondrial and cell membrane viability, in alveolar macrophages cells after 24 h of exposure to Ag NPs (NPs, Ag-15 nm, Ag-30 nm, and Ag-55 nm), quantifying ROS production [103]. In this work, MTT and LDH analysis indicated the size-dependent cytotoxic effects following Ag NPs exposure demonstrating how only Ag-15nm induced a significant increase of ROS level. The authors suggested different hypothesis to justify the experimental evidence: the reduction of the macrophages abilities to protect themselves from ROS caused by the total cell –SH reactive nature, the stronger ROS generation due to the possible unquenching of Ag-15 nm by glutathione (GSH), or the activation of apoptosis pathways. Previously, Hussain et al. demonstrated how cytotoxicity of Ag (15, 100 nm) in BRL 3A rat liver cells is likely to be mediated through oxidative stress: increase in ROS levels is related to a significant depletion of GSH level and reduced mitochondrial membrane potential [101]. In vitro effects provoked by exposure to PVP-coated Ag NPs (~70 nm) and Ag^+^ ions released by solution of AgNO_3_ were evaluated by Foldbjerg et al. on two different human cells line: monocytic cell line (THP-1) and alveolar cell line (A549) [104,105]. Study on THP-1, conducted by flow cytometric assay, showed how the induction of cell apoptosis and necrosis depended on concentration and exposure time. In addition, the drastic increase in ROS production, detected after 6–24 h, highlighted that oxidative stress plays an important role in cytotoxicity induced by Ag NPs and Ag^+^ [104]. The dose-dependent cellular toxicity caused by Ag NPs and Ag^+^ was also confirmed in A549 cells, and a strictly correlation between the levels of reactive oxygen species (ROS) and mitochondrial dysfunctions or apoptosis was demonstrated. In addition, the role of Ag compounds as ROS-induced genotoxicity was suggested by increased bulky DNA adduct amount after Ag exposure [105]. Hsin et al. [100] showed that the apoptotic effect induced by nano Ag exposure was a mitochondria-dependent mechanism. Specifically, this effect was reported for NIH3T3 mouse fibroblast, A10 rat vascular smooth muscle and HCT116 human colon cancer cells treated with Ag NPs (<100 nm) for 72 h at increasing concentration (from 0.5 ng/mL to 0.5 mg/mL). The key role played by oxidative stress in cytoxicity induced by nano Ag was also demonstrated in human hepatoma HepG2 cells [106]. Kim and co-workers investigated the toxicity events due to 24 h’ exposure to Ag^+^ and Ag NPs (from ~5 nm to 10 nm). The most cytotoxic effect was shown for free Ag^+^ ions in comparison with Ag NPs, as suggested by metal-responsive metallothionein 1b (MT1b) mRNA expression. Nevertheless, intracellular oxidative stress level was increased also following Ag NPs treatment. However, the use of the antioxidant *N*-acetyl-l-cysteine (NAC) prevented Ag toxicity and DNA-damage in HepG2 cells. The authors concluded that the oxidative stress was the first phenomena involved in Ag NPs cytotoxicity and it was independent of Ag+ ions adverse effects. Avalos et al. [107] investigated cytotoxicity induced to Ag NPs, having two different size: 4.7 nm and 42 nm, on normal human dermal fibroblasts. Smaller particles were more toxic than larger ones, as confirmed by MTT and lactate dehydrogenase (LDH) assays. In both case, the preventive addition of NAC strongly reduced the adverse effects of nano Ag exposure. Foldbjerg et al. [108] performed a systematic study by microarray technique on altered A549 cell transcriptome upon 12.1 µg/mL Ag NPs and 1.3 µg/mL Ag+ stimulation at two different time points (24 h and 48 h). They found that Ag NP altered the regulation of more than 1000 genes, whereas Ag+ only 133: different gene members of the metallothionein, heat shock protein, and histone families were upregulated. In addition, Ag+ and Ag NP treatment influenced cell cycle progression and ROS production. The authors concluded by suggesting that the cellular response to Ag^+^ was faster but less stable than Ag NP treatment. At the same time, Piao et al. [109] demonstrated that the Ag-induced toxicity in human Chang liver cells was higher after free Ag^+^ compared with NPs, having size of ranging from 5 nm to 10 nm. After 24 h of Ag exposure, ROS level increased and concurrently the GSH decreased. This elicited damage against various cellular components, such as DNA breaks, lipid membrane peroxidation, and protein carbonylation. In addition, alteration of mitochondrial membrane potential was detected by cytochrome c release from the mitochondria, resulting in the activation of caspases 9 and 3. Finally, the apoptotic effect was exerted via the activation of c-Jun NH(_2_)-terminal kinase (JNK) causing the formation of apoptotic bodies and DNA fragmentation. The DNA damage, cell cycle arrest and apoptosis events were observed in human Jurkat T cells by Eom et al. [110]. These effects were induced by activation of p38 mitogen-activated protein kinase through nuclear factor-E2-related factor-2 and nuclear factor-κB signaling pathways. Nishanth et al. [111] examined the inflammatory responses of RAW 264.7 mouse macrophages due to exposure at different time points of many kinds of NPs, such as Ag at concentrations of 5 μg/mL, having different size (~15 nm, ~40 nm). This study reveals the high propensity of Ag NPs in inflammation induction, suggested by a significant increase in IL-6, reactive oxygen species (ROS) generation, nuclear translocation of nuclear factor-κB (NF-κB), and tumor necrosis factor-alpha (TNF-α) expression. AshaRani and colleagues [112] explored the potential molecular mechanisms involved in Ag NPs toxicity in normal human lung cells (IMR-90) and human brain cancer cells (U251). In their work, it was demonstrated that nano Ag interacts with cytosolic proteins, which were adsorbed on nanoparticle surfaces, influencing the gene and protein expression profiles. In particular, in the two cell lines, Ag NP exposure provoked downregulation of both cyclin B and cyclin E, which were involved in cell cycle progression, and many DNA damage response/repair factors (XRCC1 and 3, FEN1, RAD51C, RPA1). Moreover, apoptosis pathway was activated by down regulation of p53 and caspase 3 cleavage. Finally, the Ag NPs triggered an inflammatory response through IL-8 and IL-6 cytokines secretions, and macrophages stimulations. Many studies suggest that surface modifications of Ag NPs influenced their interactions with cellular components. Ahamed et al. [113] reported that polysaccharide-coated and -uncoated Ag NPs induced genotoxic effects in two mammalian cell lines: mouse embryonic stem (mES) cells and mouse embryonic fibroblasts (MEF). The different chemical surface functionalization induced a different effect in terms of toxicity: a major damage of DNA was obtained in coated nanoparticles. Even so, the annexin V protein expression and MTT assay showed a decrease of cell viability in both NPs types. Chichova and co-workers [114] analyzed the effects on rat liver mitochondrial oxidative phosphorylation due to exposure to Ag NPs coated with polysaccharide starch (Ag NPs/Starch, Dav = 15.4 ± 3.9 nm) and trisaccharide raffinose (Ag NPs/Raff, Dav = 24.8 ± 6.8 nm). Both types of Ag NPs showed decoupling effects on intact mitochondria through the alteration of ATP synthase and ATPase activities, suggesting their ability to cross the inner mitochondrial membrane. De Matteis et al. [37] investigated the mechanism of toxicity induced by citrate-capped Ag NPs with a hydrodynamic diameter of around 20 nm in HeLa and A549 cells at concentrations of 0.06, 0.3 and 0.6 nM after 48 and 96 h. The authors firstly confirmed the negative effects on cell viability, ROS production, apoptotic pathway stimulation and DNA damage and after, they investigated the in situ degradation of Ag NPs using a fluorescent probe. They found an intracellular “heavy metal type” mechanism due to ionization of NPs in Ag^+^ in lysosomes and the consecutive release in the cytosol, which explains the stronger toxicity behavior of Ag NPs. In addition, the activation of metallothioneins upon Ag NPs demonstrated a link between ions and cell death (Figure 3b,c).

## 4. AgNPs and Theranostics

While the early efforts in nanomedicine were focused on improving the properties of already available therapeutic and diagnostic modalities, more novel strategies have emerged through the supramolecular assembly of simpler components, by means of nanoscale engineering [115,116]. Theranostics is a new technological field which develops molecular diagnostics integrated with targeted therapeutics. It comprises nanosize structures, combining targeting properties, i.e., control of the spatial and temporal release of therapeutic agents, and monitoring modalities for the response to the treatment, and assessing the effectiveness of these agents [116]. Because of its properties, theranostics increases drug efficacy and safety, and therefore it is of great interest in cancer research, becoming one of the key topics in this field [117,118,119,120]. Nowadays, much research effort has been made in order to synthetize anticancer drugs and design proper vehicles, guiding them more precisely to tumor cells and away from sites of toxicity, optimizing drug concentration and limiting size-adverse effects [117]. Moreover, an imaging function is often added to delivery vehicles by attaching contrast agents for use in non-invasive methods of imaging, including X-ray-based computer-assisted tomography (CT), positron emission tomography (PET), single photon emission tomography and magnetic resonance imaging (MRI) [121]. With respect to conventional modalities, nanoscale particles and nanovehicles are increasingly being tested for their effects on cancer cells [122]. Multifunctional nanomaterials are used as drug-delivery agents and diagnostic tools, because they offer the possibility to deliver and release drugs, targeting cancer cells in a regulated manner and at the same time allow the detection of cancer cells with enormous specificity and sensitivity [123]. Ag NPs exhibit unique physical and chemical features that make them suitable for cancer theranostic applications. As other metallic NPs, Ag NPs have a larger surface area and area:volume ratio, which in turn enhance their catalytic activity. Owing to their nanosize, they can be vehiculated to the tumor site either by passive targeting (exploiting the enhanced permeability and retention effect), or by active targeting (by means of proper ligand surface functionalization) [124]. Ag NPs are reported to have anticancer property; furthermore, Ag NPs elicit various damaging effects on structures and functions of cells, which finally induce cytotoxicity, genotoxicity, immunological responses, and even cell death. Gonipanath et al. [125] demonstrated the cytotoxic effect of Ag NPs exerted on living cells; in fact, they found that Ag NPs induced apoptosis on cancerous HT29 as well as non-cancerous BHK21 cells. Interestingly, they observed a synergistic effect on apoptosis using uracil phosphoribosyltransferase (UPRT)-expressing cells and non-UPRT expressing cells in the presence of the drug fluorouracil (5-FU), suggesting that Ag NPs, combined with traditional cancer treatment modalities, enhanced their performance. By means of biotechnology and nanotechnology, nanomaterials could be properly bioconjugated, exhibiting new features in tumor treatment [126,127]. Proper functionalized nanomaterials could affect cell growth and viability based on the size, capping or coating materials, color, surface chemistry, and dose [128]. Starting from these observations, Ag NPs were often used in combination with polymers for the delivery to cancer cells. Sanpui et al. reported the synthesis of a chitosan (CS) nanocarrier (NC)-based delivery of Ag NPs able to induce apoptosis at very low concentrations of the NPs in human colon cancer cells (HT 29) [129]. They demonstrated that Ag CS NCs induced the production of intracellular ROS also at low concentration, compared to the use of free Ag NPs. 

**Figure 4:**
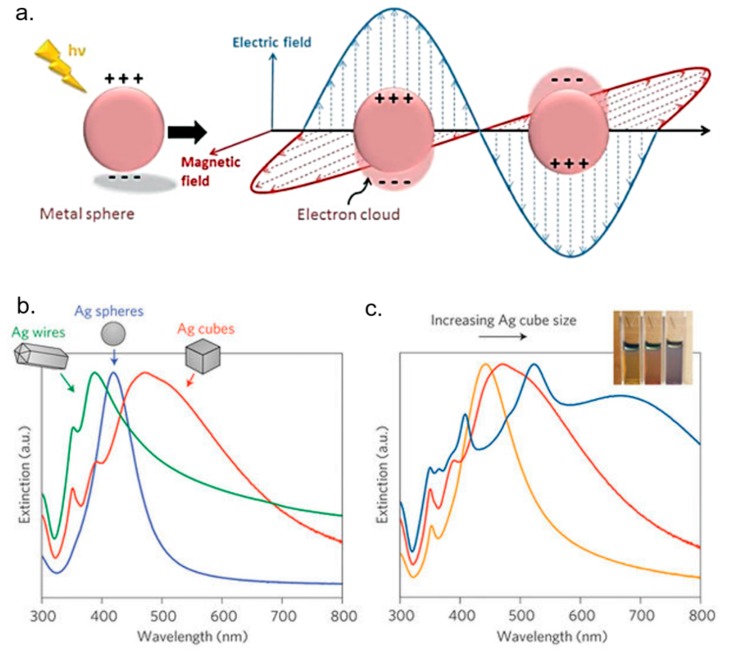
(**a**) Schematic representation of a localized surface plasmon resonance (LSPR), reproduced from [153] with permission of The Royal Society of Chemistry. (**b**,**c**) The change of shape and size exhibited different extinction properties. Adapted from [154] with permission from Macmillan Publishers (Nature Materials), 2015.

Other experimental research has proved the synergistic effect of Ag NPs and polymers concerning cancer treatment. For example, PVP-coated Ag NPs inhibited the growth of acute myeloid leukemia (AML), inducing a cytotoxic effect due to the production of reactive oxygen species (ROS), losses of mitochondrial membrane potential and DNA damage [130]. The same molecular mechanisms were reported for starch-coated Ag NPs, which were studied in normal human lung fibroblast cells (IMR-90), and human glioblastoma cells (U251) showing anticancer properties [131]. In a recent study, Liang et al., investigated the effect of Ag NPs on human glioma U251 cells and its role in the combinational use with Temozolomide (TMZ), an imidazotetrazine derivative of the alkylating agent dacarbazine, against glioma cells. They found that Ag NPs could have a potential application in enhancing chemotherapy for glioma. In fact, Ag NPs showed dose-dependent cytotoxicity on U251 cells owing to their ability to enhance the drug-sensitivity of TMZ on U251 cells [132]. Protein-conjugated Ag sulfide nano-particles, nano-rods and nano-wires were reported to inhibit the C6 glioma cells and human hepatocellular carcinoma Bel-7402 cells significantly; moreover, the effect of nano-crystals on tumor cells was crystal size-dependent [128]. Among the modalities used for reached tumor sites, targeted therapy has recently become the most attractive strategy for cancer treatment. Locatelli et al. synthetized multifunctional nanocomposites consisting of polymeric nanoparticles (PNPs) containing two cytotoxic agents (the drug alisertib and Ag NPs), conjugated with a chlorotoxin, an active targeting 36-amino acid-long peptide that specifically binds to MMP-2, a receptor overexpressed by brain cancer cells. They demonstrated the strong toxicity ability of these composite nanostructures in a human glioblastoma-astrocytoma epithelial-like cell line (U87MG) [133]. Not only chemistry synthetized Ag NPs elicited cytotoxic effects on cancer cells, but also biologically derived Ag NPs showed anticancer properties [134]. In fact, the anticancer property of bacterial (B-Ag NPs) and fungal extract-produced Ag NPs (F-Ag NPs) was demonstrated in human breast cancer MDA-MB-231 and MCF-7 cells [135,136,137]. Plant extract-mediated synthesis of Ag NPs exhibited more pronounced toxic effects in human lung carcinoma cells (A549) compared to non-cancer cells such as human lung cells, indicating that Ag NPs could target cell-specific toxicity. This mechanism could be due to the typical acidic pH value in cancer cells [138]. The interesting ability of Ag NPs to selectively affect cell viability was also confirmed by other experimental evidenc. Zureberek et al. [139] investigated the role of respiration in Ag NP-induced oxidative stress. Considering that cancer cells rely on glucose as the main source of energy supply, the authors demonstrated that glucose availability was strictly related to NP toxicity. They found that Ag NPs induced dose-dependent generation of H_2_O_2_. In addition, Ag NP toxicity for the cells maintained in the low-glucose medium was significantly lower compared to cells growing in the high-glucose concentration. This result indicated that scarceness of glucose supply resulted in upregulation of the endogenous antioxidant defense mechanisms, which in turn affected ROS generation and toxicity induced by Ag NPs. Moreover, because hydrogen peroxide is continuously formed at micromolar levels and participates in redox homeostasis [140], He and co-workers [141] studied the interaction between H_2_O_2_ and Ag NPs, examining the effects of Ag NPs upon generation of ROS and oxygen over a physiologically relevant pH range. Their results demonstrated the alteration of the balance between hydroxyl radicals, oxygen and hydrogen peroxide influenced the effects of Ag NPs. In addition, Ag NPs [142] are known to strongly interact with electromagnetic radiation (i.e., photons) [143,144] resulting in a localized surface plasmonic resonance (LSPR) [145,146] (Figure 4a). The local electric fields generated on the NP surfaces are the product of enhanced light-matter interaction, occurring only at specific resonant frequencies [147,148]. The elevated fields raise a high number of electron–hole pairs, which in turn can induce photochemical transformations [149]. However, the plasmonic resonance of conventionally spherical Ag NPs is restricted only in the visible range of the spectrum (400–550 nm) [150], and its employment in biological tissues is thus rather limited because of the high tissue-mediated visible light absorption [151]. Ag exhibits the highest efficiency of plasmonic excitation among the three metals displaying plasmonic resonance (Ag, Au, and Cu) [152]. In addition, the plasmonic peak can be tuned, varying the shape and sizes of Ag-based nanomaterials (Figure 4b). 

**Figure 5:**
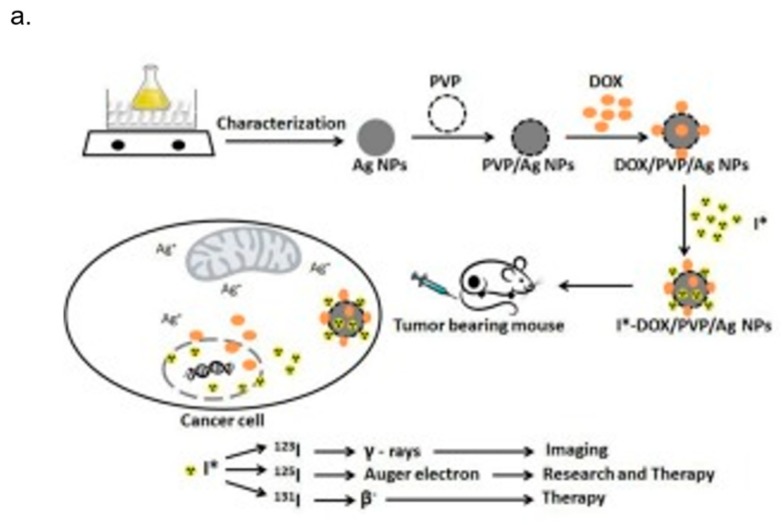

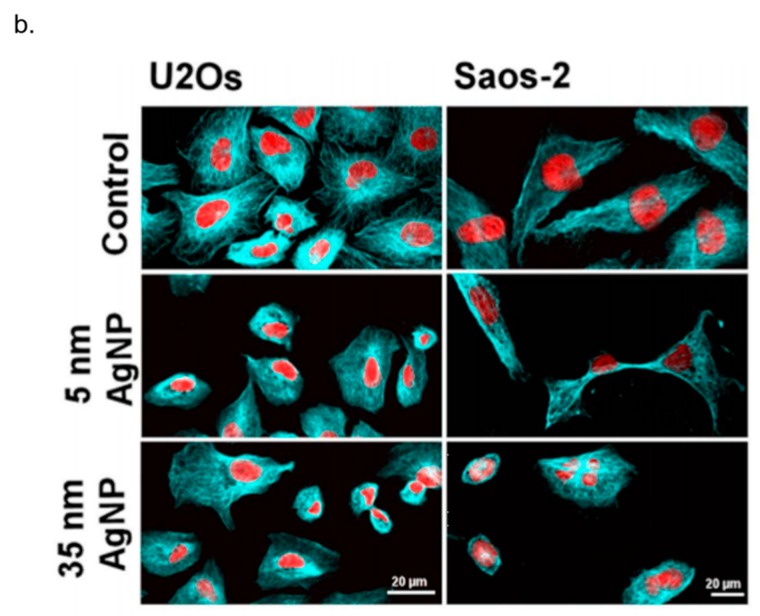
(**a**) AgNPs with PVP coating and Doxorubicin (DOX) loaded polymeric NPs (DOX/PVP/Ag NPs) AND iodine-125 isotope to tracking in vivo the NPs after intravenously injection in normal and solid tumor bearing mice. NPs specifically targeted tumor site for a long period of time making Ag-based NPs as tumor-specific agents for both diagnostic and therapeutic applications. Reprinted from Publication [156] with permission from Elsevier, 2017. (**b**) Confocal images showed the effect of AgNPs on tubulin by apoptotic events induction in U2Os and Saos-2 cells. Adapted from [158] with permission of Springer Nature, 2016.

Mukherjee et al. developed green synthesized Ag NPs with multifunctional activities, using *Olaz scandens* leaf extracts. Ag NPs exhibited anticancer activity against different cancer cells (A549: human lung cancer cell lines, B16: mouse melanoma cell line and MCF7: human breast cancer cells). In addition to that, they were biocompatible to rat cardiomyoblast normal cell line (H9C2), human umbilical vein endothelial cells (HUVEC) and Chinese hamster ovary cells (CHO). At the same time, Ag NPs showed bright red fluorescence, which could be used to detect the localization of drug molecules inside cancer cells. For these reasons, Ag NPs could act not only as anticancer drugs but also as drug delivery vehicle and imaging facilitator [155]. Farrag et al. [156] functionalized Ag NPs with PVP and antitumoral drug Doxorubicin (DOX) to quickly track by ^125^I isotope their localization in the tumor site. They showed the double use of this Ag vector, which is both diagnostic and therapeutic (Figure 5a). In another study, Boca-Farcau et al. [157] developed newly synthesized chitosan-coated Ag nanotriangles (Chit-Ag NTs) with strong resonances in near infrared (NIR), investigating their ability to operate as photothermal agents against a line of human non-small lung cancer cells (NCI-H460). By the hyperthermia experiments, they found that the rate of cell mortality in the presence of Chit-AgNTs was higher than in the presence of thiolated poly(ethylene) glycol capped gold nanorods (PEG-AuNRs) (a common hyperthermia agent used as reference). No destructive effects were detected on the control sample (cells without nanoparticles) under identical irradiation conditions. Kovács et al. [158] synthetized Ag NPs (5 and 35 nm) to test their ability to kill tumor suppressor-deficient osteosarcoma cancer cells. They found that NPs induced apoptosis in wild-type p53-containing U2Os and p53-deficient Saos-2 cells demonstrating the potential novel chemotherapeutic approaches based on Ag NPs (Figure 5b). Schrand et al. examined the chemical and biological properties of Ag NPs of similar sizes, differing in their surface chemistry (hydrocarbon versus polysaccharide), for their potential use as biological labels in neuroblastoma cells. We observed strong optical labelling of the cells in high-illumination light microscopy. Ag NPs were able to bind to plasma membrane and be uptaken and localized into intracellular vacuoles, suggesting that they could be involved in a cell labelling procedure. The major drawbacks of this approach relied on the induction of ROS, the degradation of mitochondrial membrane integrity, and the disruption of the actin cytoskeleton system after 24 h of Ag NPs exposure [159]. The large use of Ag NPs for cancer diagnosis and treatment raises concerns over their potential short- and long-term toxicity, above all in view of the application into clinical practice. Nevertheless, information about the mechanism of cytotoxicity derived mainly from in vitro studies and the impact of Ag NPs on animal models is still unclear. As reviewed elsewhere, Ag NPs elicited many toxicological responses (including effects on circulatory, respiratory, central nervous and hepatic systems) in laboratory rodents exposed to Ag NPs [160]. Results showed that the cytotoxic and genotoxic effect of Ag NPs was strictly linked to their concentration, size, surface-coating, exposure time and environmental factors [161]. Adult male C57BL/6N mice exposed to Ag NPs exhibited oxidative stress-induced neurotoxicity in brain regions [162] and other experimental evidence reported that liver and bile ducts are accumulation sites for Ag NPs [163]. Another study in zebrafish embryos suggested that the toxicity of Ag NPs was associated with bioavailable Ag^+^ [164]. More recently, Munger et al. orally administered Ag NPs in healthy volunteers and they evaluated human biodistribution, bioprocessing and possible toxicity of Ag NPs. They demonstrated that in vivo oral exposure to Ag NPs solutions did not produce clinically severe changes in human metabolic, hematologic, urine, physical findings or imaging morphology [165]. This work opened a new scenario for the use of Ag NPs in cancer theranostics in the near future.

## 5. Conclusions

In this work, we have first studied the main methods (physical, chemical and green) to synthetize Ag NPs by using bottom-up and top-down approaches. These methods are aimed at obtaining Ag NPs in a reproducible manner and with specific physico-chemical properties, suitable for various applications. We also analyzed the most recent literature concerning the toxicity mechanisms at the cellular level of Ag NPs, which makes it useful as an anti-cancer tool, combining a good intracellular and intra-tissue tracking with a therapeutic effect. Future challenges are finalized to develop Ag-based anti-tumor nanomaterials for personalized medicine treatment.
